# Genetic diversity of *Plasmodium falciparum* infection among children with uncomplicated malaria living in Pointe-Noire, Republic of Congo

**DOI:** 10.11604/pamj.2019.32.183.15694

**Published:** 2019-04-12

**Authors:** Brice Pembet Singana, Pembe Issamou Mayengue, Roch Fabien Niama, Mathieu Ndounga

**Affiliations:** 1 Faculté des Sciences et Techniques, Université Marien Ngouabi, BP 69 Brazzaville, République du Congo; 2 Laboratoire National de Santé Publique, BP 120 Brazzaville, République du Congo; 3 Programme National de Lutte contre le Paludisme, Direction Générale de l’Epidémiologie de la Maladie, Ministère de la Santé et de la Population, République du Congo

**Keywords:** Plasmodium falciparum, genetic diversity, multiplicity of infection, *msp-1*, *msp-2*, Republic of Congo

## Abstract

**Introduction:**

Molecular characterization of malaria parasites from different localities is important to improve understanding of acquisition of natural immunity to *Plasmodium falciparum*, to assist in identifying the most appropriate strategies for control and to evaluate the impact of control interventions. This study aimed to determine the genetic diversity and the multiplicity of infection in *Plasmodium falciparum* isolates from Pointe-Noire, Republic of Congo.

**Methods:**

*Plasmodium falciparum* isolates were collected from 71 children with uncomplicated malaria; enrolled into the study for evaluating the therapeutic efficacy of artemether-lumefantrine combination. Both *msp-1* and *msp-2* genes were genotyped.

**Results:**

From 296 distinct fragments detected, 13 *msp-1* and 27 *msp-2* different alleles were identified. For *msp-1*, RO33 family was poorly polymorphic. The K1 family has shown the trend of predominance (41%), followed by Mad20 (35%). Comparatively to *msp-2*, 49.6% and 48.8% fragments belonged to 3D7 and FC27 respectively. Taking together *msp-1* and *msp-2* genes, the overall multiplicity of infection has been increased to 2.64 and 86% harbored more than one parasite genotype. Parasite density was not influenced by age as well as the multiplicity of infection which was not influenced neither by age nor by parasite density.

**Conclusion:**

Genetic diversity of *Plasmodium falciparum* in isolates from patients with uncomplicated malaria in Pointe-Noire is high and consisted mainly of multiple clones. The overall multiplicity of infection has been largely increased when considering *msp-1* and *msp-2* genes together. With the changes in malaria epidemiology, the use of both *msp-1* and msp-2 genes in the characterization of *Plasmodium falciparum* infection is recommended.

## Introduction

Malaria, a disease mainly caused by *Plasmodium falciparum*, remains a public health concern. Although massive interventions deployed in sub-Saharan Africa have reduced the global malaria morbidity and mortality at 212 million and 429,000 deaths respectively in 2015, some sub-Saharan Africa region had provided partial data regarding the impact of certain interventions [1]. In the Republic of Congo, malaria is still the leading cause of attendance in health facilities. The latest estimations from the National Malaria Control Program indicate that clinical malaria account for 47.9% of all outpatient consultations in public hospitals, 64.8% of hospital admissions and 18.4% of deaths [2]. The high levels of resistance of *Plasmodium falciparum* to chloroquine as well as the inefficacy of sulphadoxine-pyrimethamine and amodiaquine either singly or in combination for the treatment of uncomplicated malaria have been well documented [3-6]. Thus, the Republic of Congo has changed its anti-malarial drug policy for treating uncomplicated malaria to artemisinin-combination therapies (ACTs) in 2006 [7]. Studies aiming to evaluate the efficacy of these combinations have been conducted mainly in Brazzaville [8-10]. Only one was done in Owando, located in the north part of the Republic of Congo [11]. Although the efficacy of artesunate-amodiaquine and artemether-lumefantrine is still high as reported in these previous studies, it is important to extend the efficacy assessment as well as to evaluate the impact of these combinations on the malaria parasite population dynamic and the multiplicity of *Plasmodium falciparum* infection (MOI) in other localities, nine years after the implementation of artesunate-amodiaquine and artemether-lumefantrine in Republic of Congo.

Genetic diversity of circulating *Plasmodium falciparum* strain, the occurrence of variant forms of the parasite in different geographic areas and variation of MOI after recombination between genetically distinct gametocytes, constitute the major obstacles to the design of a malaria vaccine [12, 13]. Consequently, these two parameters allow characterization of malaria parasites in human populations and improve understanding of acquisition of natural immunity to *Plasmodium falciparum* and would assist in identifying the most appropriate strategies for control and also to evaluate the impact of control interventions [14, 15]. The merozoite surface protein-1 *(msp-1)* and merozoite surface protein-2 *(msp-2)* are asexual blood stage antigens that are considered prime candidates for the development of malaria vaccine, and also suitable markers used extensively to identify genetically distinct *Plasmodium falciparum* parasite sub-populations in many malaria endemic countries as well as to distinguish recrudescence to re-infection in anti-malarial drug trials and efficacy [16-18]. It has been proposed that with the change of malaria epidemiology, both *msp-1* and *msp-2* allele frequency and genetic diversity should be monitored regularly to ensure the reliability of the PCR (polymerase chain reaction)-adjusted treatment outcome [18]. To our knowledge, in the Republic of Congo, after the introduction of artemisinin combination therapy (ACTs), the most epidemiological studies on *Plasmodium falciparum* genetic diversity have been conducted in Brazzaville [15, 19]. However, only one has been done in one health facility in the city of Pointe-Noire, the second largest city in the country, using *msp-2* gene as marker [20]. Conversely, there is no information on *msp-1* diversity in Pointe-Noire. During the study aiming the efficacy assessment of artemether-lumefantrine combination in Pointe-Noire in 2015, we proposed to determine the genetic diversity of *Plasmodium falciparum* in the recruited population using both *msp-1* and *msp-2* markers.

## Methods

### Study area

The present study was conducted at the “Centre de Santé Intégré de Mbota” located in the eastern part of the city of Pointe-Noire, the economic capital of the Republic of Congo, separated by 510km from Brazzaville. Two rainy seasons are observed each year with the main one during the months of February to May, and a short one from October to December. Malaria endemicity is high with perennial transmission and peaks during the rainy season, which normally runs from October to May [3] and malaria infection is primarily due to *Plasmodium falciparum*.

### Study population and blood samples collection

Infected blood with *Plasmodium falciparum* was collected from children under 13 years old enrolled into the study (study ACTRN12615001110572) for evaluating the therapeutic efficacy of artemether-lumefantrine combination from September to November 2015. Diagnostic of *Plasmodium falciparum* was confirmed by light microscopy on thick blood smears. Malaria parasites were quantified against 200 leucocytes. Parasite density was calculated for each patient assuming an average of 8,000 leucocytes per µL of blood using the proposed method of the WHO [21]. Before treatment, blood sample from each patient was blotted on the Watman filter paper (3MM CHR), dried and transferred to the “Laboratoire National de Santé Publique” in Brazzaville, where isolation of DNA and genotyping were performed. The clinical study on artemether-lumefantrine efficacy was approved by the institutional “Comité d’Ethique de la Recherche en Sciences de la Santé” (N° 038/DGRST/CERSSA in June 2015) of the Congolese Ministry of Research and the Ministry of Health and Population of Republic of Congo.

### Extraction of parasite DNA

Genomic DNA was extracted from samples collected on the Watman filter paper by QIAamp DNA mini Kit (Qiagen, Hilden, Germany) according to the manufacturer’s instruction. Extracted DNA was stored at -20°C until use.

#### Plasmodium falciparum msp-1 and msp-2 genotyping

Samples genotyping of *Plasmodium falciparum* was performed using the nested polymerase chain reactions (PCRs) technique. The merozoite surface protein-1 *(msp-1)* and merozoite surface protein-2 *(msp-2)* genes in their highly polymorphic loci, namely *msp-1* block 2 and *msp-2* central region were used as markers for this genotyping as described previously [16, 17, 22, 23]. PCR amplification was performed following a 2-step amplification procedure, in which the initial amplifications were followed by individual nested PCR reactions using specific primers for K1, Mad20 and RO33 allelic families for *msp-1*, and FC27 and 3D7 allelic families for *msp-2* (Table 1). Allelic specific and DNA free negative controls were included in each step of the reaction. Five microliters of each of the PCR products were loaded on 2% agarose gel (PeqLab, Erlangen, Germany), stained with ethidium bromide, separated by electrophoresis and visualized under ultraviolet trans-illumination. Individual alleles were identified by fragment length and by the corresponding allele-specific primers used and the size of the PCR products were estimated using 100bp DNA ladder marker (Invitrogen, Karlsruhe, Germany). The size polymorphism in each allelic family (Table 1), assuming that one band represented one amplified PCR fragment derived from a single copy of *Plasmodium falciparum msp-1* and *msp-2* genes. Alleles in each family were considered the same fragment size was within 20 bp interval [17].

**Table 1:** Oligonucleotides sequences used for single and nested PCR of Plasmodium falciparum *msp-1*and *msp-2* genes [16], and range of fragment size of the alleles

*msp-1* gene	*msp-2* gene
Single PCR	Single PCR
A+B primers 5’AAGCTTTAGAAGATGCAGTATTGAC3’ 5’ATTCATTAATTTCTTCATATCCATC3’	1+4 primers 5’ATGAAGGTAATTAAAACATTGTCTATTATA3’ 5’ATATGGAAAAGATAAAACAAGTGTTGCGT3’
**Nested PCR**	**Nested PCR**
K1 primers 5’AAGAAATTACTACAAAAGGTG3’ 5’TGCATCAGCTGGAGGGCTTGCACCAGA3’	Non specific family (2+3 primers) 5’AACGAATTCATAAACAATGCTTATAATATGAGT3’ 5’GATGAATTCTAGAACCATGCATATGTCCATGTT3’
Ro33 primers 5’AGGATTTGCAGCACCTGGAGATCT3’ 5’GAGCAAATACTCAAGTTGTTGCA3’	FC27 primers 5’GCAAATGAAGGTTCTAATACTAATAG3’ 5’GCTTTGGGTCCTTCTTCAGTTGATTC3’
Mad20 primers 5’TGAATTATCTGAAGGATTTGTACGTCT3’ 5’GAACAAGTCGAACAGCTGTTA3’	3D7 primers 5’GCAGAAAGTAAGCCTTCTACTGGTGCT3’ 5’GATTTGTTTCGGCATTATTATGA3’
**Range of fragment size of the alleles**	
***msp-1* allelic families** Mad20 alleles:180bp to 300bp RO33 alleles : 150bp to 280bp K1 alleles:130bp to300bp	***msp-2* allelic families** FC27 alleles: 260bp to540bp 3D7 alleles:170bp to 470 bp

### Data and statistical analysis

The frequency of *msp-1* and *msp-2* allele was calculated as the proportion of allele found for the allelic family out of the alleles detected in isolates. The mean expected heterozygosity (He) value for each allelic family of *msp-1* and *msp-2* genes was calculated as described by Mohd Abd Razak *et al.* [24]. The detection of one *msp-1* and *msp-2* allele was considered as one parasite genotype. The multiplicity of infection was defined as the minimum number of *Plasmodium falciparum* genotypes per infected subject and estimated by dividing the number of amplified PCR fragments reflecting the parasite genotypes by the number of positive samples. Chi-square test was applied to compare proportions. Spearman’s rank correlation coefficients were calculated to assess association between multiplicity of infection (MOI) and geometric mean parasite density and age. Differences were considered significant if P values < 0.05.

## Results

### Characteristics of patients and parasite density

A total of 71 patients with malaria and microscopically confirmed *Plasmodium falciparum* only were enrolled in the study, with 34 (47.9%) and 37 (52.1%) being males and females respectively. Twenty-six and 45 were children aged between 1 to 4 years old and 5 to 12 years old respectively. The geometric mean parasite density was 36,699.6 parasites/µl of blood with the range of 1,435-349,700 parasites/µl (Table 2).

**Table 2:** Characteristics of patients with uncomplicated *Plasmodium falciparum* malaria in Pointe- Noire, Republic of Congo

Characteristics of patients	Values
Gender (Male/Female)	34/37
Mean age (years)	5.98 (1-12)
Group of age < 5 years (%)	26 (36.6)
Group of age ≥ 5 years (%)	45 (63.4)
Mean parasite density (parasites/µl)	36,699.6 (1,435-349,700)
Multiplicity of infection	2. 64 (1-6)
Polyclonal infections (%)	61 (86)
***%*** percentage	

#### Plasmodium falciparum genotyping of msp-1 and msp-2

**Figure 1:**
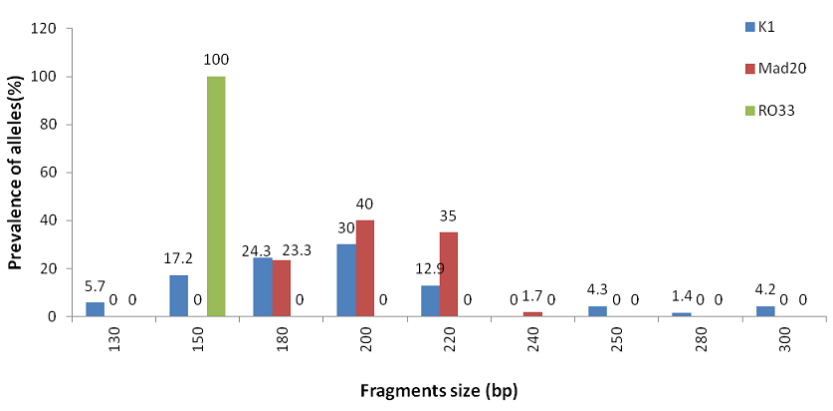
Prevalence of *Plasmodium falciparum msp-1* alleles in clinical isolates from Pointe-Noire, Republic of Congo

**Figure 2:**
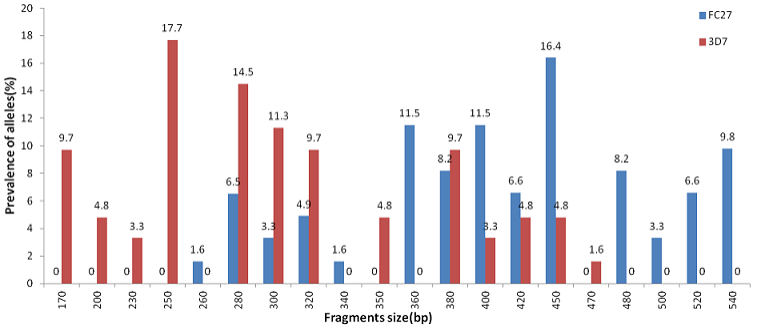
Prevalence of *Plasmodium falciparum msp-2* alleles in clinical isolates from Pointe-Noire, Republic of Congo

All 71 samples were analyzed for polymorphisms on *msp-1* and *msp-2* genes. The efficiency of *msp-1* and *msp-2* genes amplification reactions with family-specific primers were 100% and 97.2% respectively. With a total of 296 distinct fragments detected, 13 *msp-1* and 27 *msp-2* different alleles were identified. The *msp-1* gene analysis showed 70, 60, 41 fragments belonged to K1 (41% of overall detected *msp-1* alleles), Mad20 (35%) and R033 (24%) families respectively. Eight K1 type alleles (He value of 0.82), 4 Mad20 (He value of 0.68) type alleles and only 1 RO33 type allele (150 pb) were identified, with no statistical significant predominance of any specific family (Figure 1). The *msp-2* gene yielded 13 3D7 type alleles (He value of 0.92) and 14 FC27 type alleles (He value of 0.93), with no statistical significant predominance of any specific family (Figure 2). Out of 123 fragments identified, 62 fragments belonged to 3D7 (49.6% of overall detected *msp-2* alleles) and 61 belonged to FC27 (48.8%). Two genotypes (1.6%) could not be assigned to any specific family.

#### *Plasmodium falciparum* multiplicity of infection, parasite densities in relation to age

**Figure 3:**
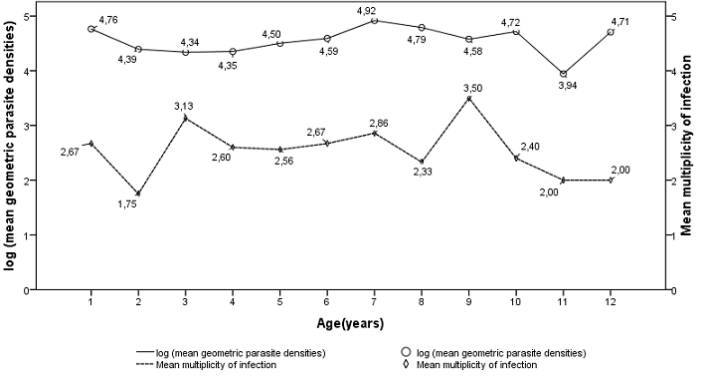
Relation between mean geometric density and multiplicity of infection with age

When considering *msp-1* and *msp-2* genes separately, the MOI was 2.40 and 1.74 respectively, while 55 (77.46%) and 34 (47.88%) of isolates contained multiclonal infection at least with 2 clones respectively. Taking together *msp-1* and *msp-2* genes, the overall MOI was 2.64 and 61 (86%) isolates harbored more than one parasite genotype. No statistical significant difference was observed in the MOI between patients with less than 5 years old (2.44) and those with the age between 5 and 12 years old (2.40) with the *p-value* = 0.972. The combinations of *msp-1* and *msp-2* different parasite genotypes were not associated with the parasite density (Table 3), as well as the MOI was not associated with parasite density (Table 4). Moreover, the age was not associated significantly with the parasite density (*p-value* = 0.381). Taking together, no statistical significant difference was observed in the parasite densities and the MOI according to the age (Figure 3).

**Table 3:** Distribution of *msp-1* and *msp-2* detected allelic families according to the parasite densities

Mean parasite densities			
*msp-1*	*msp-2*		
	FC27	3D7	FC27 + 3D7
K1	146840	118222	4466
Mad20	53295	24219	4060
RO33	113982	135877	0
K1+Mad20	36063	57954	119478
K1+RO33	157537	12077	79840
Mad20+RO33	47697	26370	5140
K1+ Mad20+RO33	60389	32979	10103

**Table 4:** Profile of multiple infections according to the parasite densities

Multiplicity of infection	Frequency	Mean parasite densities
1	10	39,981
2	22	32,092
3	27	39,057
4	8	38,964
5	3	18,879
6	1	252,595

## Discussion

The genetic diversity may be an important element for implementing malaria control strategies in the country, as elimination may influence genetic diversity [25]. In the Republic of Congo, most of the studies on the *Plasmodium falciparum* genetic diversity using merozoite surface protein genes which are considered like robust and suitable markers [22, 25, 26] have been conducted in Brazzaville, the political capital [5, 15, 17, 19, 27]. With the expanding access to ACT and current changes in malaria epidemiology, it has been encouraged to monitor regularly the *Plasmodium falciparum msp-1* and *msp-2* allele frequency-genetic diversity [18], despite some debate around their usefulness as markers of population structure [14, 28]. To our knowledge, this present study represents the first investigation of genetic diversity of *Plasmodium falciparum* populations to employ both *msp-1* and *msp-2* gene markers, 9 years after the implementation of ACTs for the treatment of uncomplicated *Plasmodium falciparum* malaria in Pointe-Noire.

Allelic specific PCR typing of *msp-1* and *msp-2* genes showed a high genetic diversity in the *Plasmodium falciparum* population in the analyzed isolates. The degree of polymorphism found in the present study is also consistent with the previous findings in the same area using microsatellites markers [29]. The *msp-2* gene has showed a high diversity as previously reported in Pointe-Noire [20]. Although *msp-1* gene was amplified in all isolates, genetic diversity still low compared to *msp-2* gene as previously described in the country [17]. With regard to *msp-1*, the presence of Mad20, K1 and R033 allelic families in the 71 isolates was noticed. RO33 family was poorly polymorphic with only one allele as reported in previous studies in Brazzaville and other countries such as Senegal, Iran, Nigeria and Brazil [13, 17, 30-32] in contrast to findings in Gabon and Côte d’Ivoire [33]. Although the K1 family has shown the trend of predominance (41%), followed by Mad20 (35%), the difference in prevalence between these free families was not statistically significant. Comparatively to *msp-2*, no predominance to any allelic family was found. Taking together *msp-1* and *msp-2*, our findings are in contrast to those reported in previous studies in Brazzaville and other countries such as Gabon, Benin, Ghana, and Ethiopia where K1 for *msp-1* and 3D7 for *msp-2* were predominantly found in isolates from uncomplicated malaria [17, 25, 33-36].

The trend observed in the prevalence of K1 and 3D7 families in the current study may be related to the limited sample size. Repeated studies including a large sample size and different sites including symptomatic and asymptomatic *Plasmodium falciparum* infection in Pointe-Noire are needed to better characterize the allelic polymorphism of *Plasmodium falciparum* in this city. Understanding the diversity of *Plasmodium falciparum* has implications for vaccine since the major obstacles to the design of an efficient malaria vaccine is the large genetic diversity of vaccine targets allowing parasites with mutated genes to escape from the host’s immune response [16]. Thus, different genetic profiles of malaria parasites even within or between countries should be taken into account in the prospect of vaccine development against *Plasmodium falciparum* for improving vaccine design.

The presence of more than one parasite genotype was detected in 86% of isolates from Pointe-Noire, with the MOI values for *msp-1* and *msp-2* genes of 2.40 and 1.74 respectively. The overall MOI value of 2.64 was found, when taking together *msp-1* and *msp-2* genes and this was higher than values obtained from Brazzaville, even before or after the introduction of ACTs [5, 17, 19, 20]. Despite the lack of entomological data from Pointe-Noire and Brazzaville, the number of clones coinfecting a single host can be used as an indicator of the level of malaria transmission or the level of host acquired immunity [12, 28, 33, 37]. Therefore, the discrepancies on the MOI may suggest the different level of malaria transmission between these two cities, with Brazzaville being more urbanized. Studies conducted in Mali and Malawi have confirmed lowest levels of MOI and proportions of polygenomic infections in areas exhibiting low malaria transmission, namely in the urban sites and highest levels where malaria transmission is almost perennial [38, 39]. Multiple parasite types in a single human host have been suggested to lead to cross fertilization, meiotic recombination and generation of new strains during the developmental stage in the mosquito [12, 13, 40]. This supports the fact that both MOI and polymorphism are high in Pointe-Noire. Since no data are available on the MOI in Pointe-Noire before the introduction of ACT, it seems difficult to evaluate the impact of this measure on the MOI in this city. The MOI was influenced neither by age nor by parasite density in this population in contrast with the previous findings in Brazzaville, Tanzania and Sudan [17, 26, 41, 42], but concordant with those in Ethiopia and Mali [25, 39]. Therefore, regardless of the parasite densities, and the fact that the samples collection has been done during the peak malaria transmission, the prevalence of multi clonal infections affected all the two age groups.

## Conclusion

Genetic diversity of *Plasmodium falciparum* in isolates from patients with uncomplicated malaria in Pointe-Noire, Republic of Congo is high and consisted mainly of multiple clones. By considering *msp-1* and *msp-2* genes together, the overall multiplicity of infection has been largely increased. Thus, with the changes in malaria epidemiology, this study supports the use of both *msp-1* and *msp-2* genes in the characterization of *Plasmodium falciparum* infection, when the MOI is considered like one of the major parameters to be evaluated for malaria control interventions.

### What is known about this topic

- Malaria is still the leading cause of attendance in health facilities in many sub-Saharan countries;
- *Plasmodium falciparum* genetic diversity would assist in identifying the most appropriate strategies for control and also to evaluate the impact of control interventions as well as changes in malaria epidemiology;
- Little data available regarding the *Plasmodium falciparum* genetic diversity and the MOI using both *msp-1* and *msp-2* as molecular markers, particularly in Pointe-Noire, the Republic of Congo.

### What this study adds

- Genetic diversity of *Plamosdium falciparum* is high and consisted mainly of multiple clones in Pointe-Noire;
- Increase MOI when *msp-1* and *msp-2* are used together as molecular markers;
- The epidemiological trend of malaria seems to be different between Pointe-Noire and Brazzaville in the Republic of Congo.

## Competing interests

The authors declare no competing interests.
